# Impulsivity and Alcohol Use during Pregnancy and Postpartum: Insights from Novel Methodological Approaches within the Context of the COVID-19 Pandemic

**DOI:** 10.3390/bs13070600

**Published:** 2023-07-18

**Authors:** Sharon L. Ruyak, Melissa H. Roberts, Stephanie Chambers, Xingya Ma, Jared DiDomenico, Richard De La Garza, Ludmila N. Bakhireva

**Affiliations:** 1 College of Nursing, University of New Mexico, Albuquerque, NM 87131, USA; 2 Substance Use Research and Education (SURE) Center, College of Pharmacy, University of New Mexico, Albuquerque, NM 87131, USA; mhroberts@salud.unm.edu (M.H.R.); xinma@salud.unm.edu (X.M.); jdidomenico@salud.unm.edu (J.D.); lbakhireva@salud.unm.ed (L.N.B.); 3 Department of Family and Community Medicine, University of New Mexico, Albuquerque, NM 87131, USA; stchambers@salud.unm.edu; 4 David Geffen School of Medicine, Department of Psychiatry and Biobehavioral Sciences, University of California, Los Angeles, CA 90095, USA

**Keywords:** COVID-19, stress, impulsivity, substance use, ecological momentary assessment

## Abstract

Impaired emotion regulation and impulsivity have been linked to substance use. This study evaluated the association between emotion regulation difficulties—specifically impulsivity—and substance use within the context of the COVID-19 pandemic among pregnant (*n* = 49) and postpartum (*n* = 20) women. Participants from a prospective cohort ENRICH-2 completed a baseline phone survey of COVID-19-related experiences and impulsivity followed by a 14-day (3x/day) mobile ecological momentary assessment (mEMA) of impulsivity and substance use. Between-subject (BS) and within-subject (WS) associations for baseline impulsivity and momentary impulsivity with respect to substance use were examined using mixed effects models. At the BS level, momentary impulsivity scores that were higher than the overall group average were positively associated with subsequent momentary reports of marijuana use (β = 1.25; *p* = 0.04) when controlling for pregnancy status and COVID-19-related stress. At the WS level, momentary impulsivity scores that were higher than an individual’s average score were positively associated with subsequent reports of momentary alcohol use (β = 0.08; *p* = 0.04). This research supports the idea that impulsivity varies based on individual situations, such as stress associated with the COVID-19 pandemic, and may be an important correlate of substance use in pregnant and postpartum women. Future research might consider investigation of additional factors, which may serve to moderate or mediate the relationship between impulsivity and substance use.

## 1. Introduction

Impaired emotion regulation strategies have been identified as a key factor in vulnerability to mental health disorders, including in the population of pregnant women [1,2]. Dysregulation in these strategies interferes with an individual’s ability to engage in appropriate behavior in response to stressful situations, possibly resulting in impulsive, unplanned actions [2,3]. Furthermore, emotional states are unstable and change rapidly [2]. Impulsivity, a specific dimension of emotion regulation, varies within individuals on a daily basis [4]. Importantly, individuals with emotion regulation difficulties [5]—and impulsivity in particular [6]—are more likely to experience substance use disorders (SUD).

### 1.1. COVID-19 Stress

The incidence of the novel severe acute respiratory syndrome coronavirus 2 (SARS-CoV-2)—and the associated coronavirus disease 2019 (COVID-19)—was declared a global pandemic in March 2020 [7]. Historically, events such as disasters or epidemics are associated with trauma and consequent adverse behavioral health outcomes, including psychosocial stress and impaired emotion regulation [8,9,10]. Emerging evidence related to the COVID-19 pandemic is consistent with these historical findings. In fact, several surveys—weighted to be representative of the U.S. population by education, ethnicity, gender, race and other factors—conducted between March 2020 and September 2022 demonstrated that 41% of U.S. adults experienced high levels of psychological distress at least once due to the pandemic [11]. Individuals aged 18 to 29 were particularly vulnerable, with 58% reporting experiences of high levels of distress at least once across the time frame of surveys [11]. Notably, women (48%) were more likely to report a serious increase in psychological distress related to the pandemic relative to men (32%) in at least one of the four surveys conducted [12].

Similar trends in psychological distress related to the pandemic have been noted in the vulnerable population of pregnant and postpartum women. A national (47 states) online survey examining the impact of the COVID-19 pandemic on pregnant women (n = 2740) revealed that more than half of respondents reported increased stress related to food insecurity (59.2%), possible loss of income (63.7%) and loss of childcare (56.3%) [13]. An overwhelming 93% of respondents reported stress related to possible infection with COVID-19 [13]. A recent study of 135 pregnant women residing in Colorado found that participants reported higher levels of depressive and anxiety symptoms, as well as higher levels of loneliness, during the COVID-19 pandemic compared to pre-COVID-19 [14]. Together, these findings highlight the concerning impact the COVID-19 pandemic has had on the U.S. population, and specifically, the vulnerable population of pregnant and postpartum women.

### 1.2. Substance Use

The use of substances during distressing situations in order to combat stress, social isolation, anxiety and uncomfortable emotions is common [15,16,17]. Consistent with these historical patterns, increases in substance use have been observed since the onset of the COVID-19 pandemic. The findings from a systematic review of evidence dating from December 2019 to November 2020 revealed a wide range of results across studies. For example, the percentage of individuals reporting alcohol consumption ranged between 21.7% and 81.4% [18]. However, there was also a sub-group of individuals, between 17% and 32%, who did not report alcohol consumption [18]. There was a clear increase in the use of other substances—including marijuana—in the general population [18]. Noted risk factors for this increased usage included isolation, fear and distress, mental health conditions and impulsivity [18]. Similar increases in substance use—including alcohol, marijuana and tobacco—to cope with the pandemic have been noted in pregnant women [19]. These findings strongly support the conclusion that the COVID-19 pandemic has contributed to the severity of the substance use crisis in the U.S.

### 1.3. Impulsivity

Emotion regulation is a multi-dimensional construct, inclusive of an individual’s awareness, understanding and acceptance of one’s emotions, the ability to refrain from impulsive behavior in the face of negative emotions and the ability to use appropriate emotion regulation strategies to meet situational goals and demands [20]. Impulsivity, **as** one dimension of overall emotion regulation [20], can be defined as a tendency toward rapid, unplanned responses to intrinsic or extrinsic stimuli with no thought of the negative consequences for self or others [21]. Notably, the concept of impulsivity may be composed of several separate personality or behavioral factors, which can be operationalized and measured through a variety of mechanisms [21,22,23]. Within the context of the difficulties in emotion regulation scale (DERS), impulsivity is conceptualized as difficulty remaining in control of behavior when faced with negative emotions [20]. Frequently, impulsivity is conceptualized as a stable individual trait, which can be compared between individuals [22,23]. However, impulsivity can also be operationalized as a momentary state behavioral response to a specific situation [23,24]. Inherent in this state conceptualization is the idea that impulse behavior varies within individuals across time [24]. The consideration of both trait impulsivity and momentary state impulsivity, which may lead to substance use, is critical [25], particularly given supporting evidence that individuals with poorer impulse control are more susceptible to SUD [5,26].

### 1.4. Momentary Assessment

Retrospective self-report questionnaires ask individuals to report on their typical behavior, as opposed to capturing dynamic behavioral states associated with a specific situation [27]. The measurement of dynamic momentary behavioral states requires the use of novel methodological approaches. Mobile ecological momentary assessment (mEMA) involves repeated sampling of an individual’s emotions and behaviors as they occur in real time, thus minimizing the potential for recall bias, and possibly representing a better method of collecting mood data than retrospective surveys [28,29]. It has been suggested that impulsive traits correspond to specific impulsive states. In two independent samples of adolescent individuals, the EMA measures of impulsive states demonstrated strong convergent and criterion validity, and variations in the responses to EMA queries reflected both between-subject and within-subject variance [27]. This suggests that the use of EMA enhances the ability to examine when and in what context certain behaviors may occur [27]. Mobile EMA has been used successfully during pregnancy to measure comorbid substance use and post-traumatic stress disorder (PTSD) [30], mood and tobacco use [31], and perceived stress and negative mood [32]. However, to date, most studies examining the associations among impulsivity and substance use have focused on the general population. Given the established role of impulsivity as a risk factor for substance use, this leaves a critical gap in the knowledge surrounding associations between momentary impulsivity and substance use in pregnant and postpartum women.

### 1.5. Objectives and Hypotheses

Because impulsivity is both a trait and a state, our design considered both between-subject (BS) variability, where relatively stable variation is compared between individuals, as well as within-subject (WS) variability, where variation within the same individual across time is examined. Specifically, we evaluated whether (a) baseline impulsivity, measured through the DERS impulse control sub-scale, or (b) momentary impulsivity, measured through repeated mEMA surveys, was associated with the risk of substance use in pregnant or postpartum women within the context of stress related to the COVID-19 pandemic. We tested the null hypothesis of no association between baseline or momentary impulsivity and subsequent substance use with an alternative hypothesis that greater baseline and momentary impulsivity would be associated with increased risk of instances of substance use.

## 2. Materials and Methods

### 2.1. Study Design and Population

This was a supplementary study using an established prospective birth cohort ENRICH-2 to examine the effect of the SARS-CoV-2/COVID-19 pandemic on adverse psychosocial outcomes in pregnant and postpartum women. The ENRICH-2 birth cohort study included four prospective study visits: one during early pregnancy (12–27.9 gestational weeks); a second visit in the early third trimester (28–32 weeks); a third visit during the hospital stay for delivery/birth; and a final assessment of infants at 6 months of age. The inclusion criteria for the parent ENRICH-2 study were (1) singleton pregnancy; (2) gestational age: 12–28 weeks; (3) delivering at the University of New Mexico (UNM) Hospital; (4) currently living in New Mexico and planning to remain for 6 months after delivery; and (5) ability to provide written consent in English. The exclusion criteria (either at the time of initial enrollment or later in the study) were (1) prenatal use of cocaine, methamphetamines, medications for opioid use disorder (methadone, buprenorphine) or MDMA (per self-report, positive urine drug tests or medical records review); (2) delivery at <35 weeks gestational age; (3) diagnosis of fetal or neonatal anomaly or severe complication.

This study employed a baseline assessment through structured phone interview to assess emotion regulation and impulse control followed by mEMA data collection of real-time impulsivity and substance use for two weeks. ENRICH-2 participants who were pregnant or had a child ≤ 18 months of age were eligible for inclusion in the supplemental study. An additional inclusion criterion included access to a smartphone. Enrollment for the supplement began in December 2020, and data collection was completed in May 2022. Therefore, this study was conducted within the ongoing, evolving context of the COVID-19 pandemic. The study was approved by the UNM Human Research Review Committee, and all participants provided written informed consent.

### 2.2. Assessments

#### 2.2.1. Coronavirus Perinatal Experiences Impact Survey (COPE-IS)

The experiences of new and expectant mothers related to the evolving COVID-19 pandemic were assessed by the COPE–IS. The COPE-IS is a 50-item questionnaire, which assesses the experiences of pregnant and postpartum individuals within the context of the COVID-19 pandemic. The questionnaire includes queries about perinatal experiences, exposures and symptoms (self and family), financial concerns (current and expected), social support, social and activity restrictions, coping and adjustment, emotions and feelings, as well as physical and mental health [33]. Data were collected on COVID-19-related stress using the following question: “How has the COVID-19 outbreak changed your stress levels or mental health?”. The responses were as follows: “worsened significantly”, “worsened moderately”, “no change”, “improved moderately”, “improved significantly”. Data were also collected on the overall level of stress related to the COVID-19 outbreak using a Likert scale ranging from 1 (*nothing*) to 7 (*extreme*).

#### 2.2.2. COVID-19 Community Response Survey (CRS) Module 10—Substance Abuse

Changes in substance use patterns (alcohol, tobacco/nicotine and marijuana) secondary to the pandemic were assessed through administration of a modified version of the CRS Module 10 using a structured phone interview. Participants were queried about the following: (1) for the past 30 days, the number of days they drank, used tobacco or other forms of nicotine, or used marijuana or other forms of THC, and the quantities (number of drinks for alcohol, number of puffs for cigarettes and units of THC for marijuana) of substance used; and (2) during the pandemic, whether they used alcohol, tobacco/nicotine or marijuana/THC more than usual or less than usual.

#### 2.2.3. Difficulties in Emotion Regulation Scale (DERS) Impulse Sub-Scale

Difficulties in emotion regulation were assessed at baseline through administration of the DERS via phone interview. The DERS is a 36-item self-report measure of subjective emotion regulation [20]. The DERS has multiple components and assesses how a participant relates to their emotions in six areas: (1) non-acceptance of emotional responses, (2) difficulty engaging in goal-directed behavior, (3) impulse control difficulties, (4) lack of emotional awareness, (5) limited access to emotion regulation strategies and (6) lack of emotional clarity. The DERS impulse sub-scale, a component of the DERS scale, was used to assess baseline impulsivity. The DERS impulse sub-scale assesses an individual’s difficulties remaining in control of their behavior when experiencing negative emotions. The sub-scale contains 6 items (“I have experienced my emotions as overwhelming and out of control”, “When I am upset, I become out of control”, “When I was upset, I felt out of control”, When I am upset, I feel like I can remain in control of my behaviors”, “When I am upset, I have difficulty controlling my behaviors”, and “When I am upset, I lose control over my emotions”) on a 5-point Likert scale ranging from 1 (*almost never*) to 5 (*almost always*). One item is reverse scored, and the DERS impulse sub-scale score range is 6 to 30. Higher scores on the scale are indicative of greater difficulties with impulse control.

#### 2.2.4. Mobile Ecological Momentary Assessment (mEMA) Surveys

Participants were assisted in the installation of an mEMA application (Ilumivu, Inc., Cambridge, MA, USA, https://ilumivu.com) on their smartphone. Over a 14-day period, participants were cued to complete 3 mEMA surveys per day (morning, afternoon and bedtime), resulting in a maximum of 42 surveys per participant. An alert was sent at pre-determined times to remind participants to complete the survey. All surveys were time-stamped at initiation and at completion by the EMA application. Data were stored locally on the participant’s phone until they were within the cell or WiFi range when automatic download to a secure server occurred.

The DERS impulse sub-scale, as described above, was used in the mEMA surveys to assess momentary impulsivity since the last mEMA session (subsequently referred to as momentary impulsivity). At each survey point, participants were also asked to report substance use since the last mEMA session via survey questions, which queried about the number of drinks for alcohol, number of puffs for cigarettes and units of THC for marijuana. Substance use responses were converted to binary variables of any use vs. none for analysis.

### 2.3. Statistical Analyses 

Sociodemographic and medical characteristics (age, ethnicity, race, marital status, education, employment status, pregnancy status) were summarized by means and standard deviations (SD) for continuous variables and number (percentage) for categorical variables. Baseline survey responses were compared among pregnant and postpartum women. Multi-variable logistic regression was used to examine the association between self-reported baseline impulsivity (DERS impulse score) and substance use in the past 30 days after adjusting for pregnancy status and COVID-19-related stress. The baseline DERS impulse score was grand-mean-centered to examine BS (to reflect whether the subject-level score was higher or lower than other individuals) association.

To examine the associations between momentary impulsivity and subsequent substance use, a dataset consisting of mEMA responses to the momentary impulsivity score, linked with responses to the next mEMA survey, was created. Each participant had 42 survey requests (3 surveys for each of the 14 days) for a total 2898 possible study observations (69 participants × 42 surveys). The last momentary impulsivity survey request was not used, since there was no subsequent substance use information, reducing the possible study observations to 2829. In this dataset, there were 2248 momentary impulsivity survey responses (79.5%). There were 49 observations with no subsequent substance use response, resulting in a final dataset of 2199 matched momentary impulsivity scores and subsequent substance use responses. The association between mEMA momentary impulsivity response and a subsequent report of substance use was examined using a mixed effects logistic regression model for longitudinal data, with reported substance use as the dependent variable and momentary impulsivity score, pregnancy status and COVID-19-related stress as independent variables. To assess independent BS and WS associations, two momentary impulsivity score values were included in the model: (1) a person-mean-centered value (WS) and (2) a grand-mean-centered value (BS). The mixed effects model used a random intercept for subjects. As a sensitivity analysis, an alternative mixed effects logistic regression model for longitudinal data was constructed, with reported substance use as the dependent variable and momentary impulsivity score modeled as a binary variable. An optimal cut-off value for the impulsivity score was identified by comparing models using cut-off values of 8, 9, 10 and 11. The model with the lowest Bayesian information criterion (BIC) and Akaike information criterion (AIC) values was selected, which corresponded to the cut-off value of 1.

Statistical analyses were conducted using SAS statistical software (Cary, NC version 9.4). Analyses were two-tailed with an alpha level of 0.05 used to determine statistical significance; however, significance using an alpha level of 0.10 was also reported.

## 3. Results

A total of 76 ENRICH-2 participants (53 pregnant and 23 postpartum) were enrolled in the supplemental study. Fifty-three pregnant participants were recruited between parent study visits 1 (second trimester) and 3 (delivery). Postpartum participants were recruited between 3 and 18 months postpartum (13.7 ± 4.3 months). Of the 76 participants, all completed the baseline phone survey, and 69 participants completed more than one mEMA survey (range: 3–42 mEMA responses). Table 1 shows the characteristics of the 69 participants with both assessments. The mean maternal age was 30 years. Slightly more than half of the participants were Hispanic/Latina (54.9%) and had a college or professional degree (52.1%). The majority were married/cohabitating (81.7%) and were employed (66.2%). In response to the baseline COPE-IS survey, the majority of participants reported that the COVID-19 pandemic worsened their mental health either moderately (62.3%) or significantly (23.2%). Furthermore, on a scale of 1 (nothing) to 7 (extreme), almost 64% of participants rated their overall level of stress related to COVID-19 at 4 or greater.

The results of logistic regression examining the association between baseline impulsivity scores and baseline report of any substance use in the past 30 days (yes/no), controlling for pregnancy status and COVID-19-related stress, are displayed in Table 2. Although not significant, there was a trend toward a positive association between individuals with higher average baseline impulsivity score and any reported alcohol use in the past 30 days (β = 0.24; *p* = 0.08). Significant associations were not observed between baseline impulsivity scores and any use of marijuana or nicotine in the past 30 days (*p* > 0.10).

The results of multi-level models examining the association between momentary impulsivity and substance use are displayed in Table 3. At the WS level, there was a positive association between momentary impulsivity scores and subsequent momentary reports of alcohol use (β = 0.08; *p* = 0.04). At the BS level, we found a positive association between momentary impulsivity scores and subsequent momentary reports of marijuana use (β = 1.25; *p* = 0.04) when controlling for pregnancy status and COVID-19-related stress. Significant associations between momentary impulsivity and nicotine use were not observed at the BS or WS level (*p* > 0.10).

The results of logistic regression examining momentary impulsivity as a binary variable (high/low) are displayed in Table 4. In this analysis, having a momentary impulsivity score ≥10 vs. <10 was associated with almost three-times higher odds of alcohol use (OR = 2.77, 95% CI [1.28–6.00]). As mentioned before, in these models, no significant associations were noted for momentary impulsivity and marijuana or nicotine use (*p* > 0.10).

## 4. Discussion

The current study examined the associations between emotion regulation capabilities—specifically impulsivity, as measured through the DERS impulse sub-scale—and substance use in pregnant and postpartum women within the context of the COVID-19 pandemic. The findings from this study support the growing body of knowledge regarding the effect of COVID-19-related stress on the well-being of pregnant and postpartum women. Participants reported high levels of stress related to the COVID-19 pandemic, and a large portion of participants reported that the COVID-19 pandemic worsened their mental health. Importantly, the study findings show that momentary reports of impulsivity were associated with reports of alcohol and marijuana use in pregnant and postpartum participants.

### 4.1. Stress Related to the COVID-19 Pandemic

It is not surprising that the majority of study participants reported that the COVID-19 pandemic worsened their mental health. Additionally, our results show high levels of self-reported stress related to the COVID-19 pandemic in pregnant and postpartum participants. These results are similar to previous reports of the effect of disasters on pregnant and postpartum women. For example, in January 1998, the Canadian province of Quebec experienced a series of ice storms, which resulted in power outages lasting from 1 day to up to 6 weeks. Project Ice Storm was undertaken to determine the effects of the disaster on pregnant women and their unborn children. The survey results demonstrated high levels of self-reported subjective stress, with almost 17% of participants reporting symptoms consistent with potential PTSD [34]. Similarly, in 2005, New Orleans was devastated by Hurricane Katrina. The survey results from 402 pregnant women revealed that mental health problems—including symptoms of depression, anxiety, PTSD and perceived stress—were a significant concern for women living in New Orleans in the aftermath of the disaster [35]. Our results are also consistent with contemporary reports of increased rates of stress and mood disorders in pregnant and postpartum women related to the COVID-19 pandemic [36,37]. These findings lend support to the growing body of literature detailing the impact of the pandemic on this vulnerable population and further highlight the need for continued longitudinal research to inform clinical assessment and intervention to prevent adverse perinatal outcomes.

### 4.2. Trait Impulsivity and Alcohol Use

Second, our results demonstrate a trend toward a positive association between higher than average baseline impulsivity scores and alcohol use in the past 30 days in pregnant and postpartum women. At this time, there is a paucity of literature examining trait impulsivity in this population. However, there is an emerging body of evidence surrounding the broader concept of emotion regulation during pregnancy and the postpartum period [2,38]. Moreover, the evidence points to the possibility of variations in emotion regulation capabilities across major transitional stages, such as pregnancy and motherhood [39].

There is a clear association between trait impulsivity and increased substance use in the general population. In a recent study of racial–ethnic minority adolescents, the results demonstrated that several impulsivity traits, including positive urgency and sensation seeking—the facets of impulsivity measured through the urgency–premeditation–perseverance–sensation seeking–positive-urgency (UPPS-P) impulsive behavior scale—were related to alcohol use [40]. Additionally, an international (83 countries) survey examining alcohol use patterns of adults 18 years and older, before and during COVID-19 quarantine, demonstrated that the patterns of alcohol use were significantly associated with positive urgency impulsivity—a trait assessed by the short impulse behavior scale (SUPPS-P) [41].

### 4.3. State/Momentary Impulsivity and Substance Use

We also examined whether state or momentary impulsivity measured through repeated mEMA surveys was associated with increased substance use. State impulsivity is a behavioral response to a specific situation [23,34]. Therefore, state impulsivity varies within individuals across time [24]. At the WS level, we found a positive association for momentary impulsivity scores and subsequent mEMA reports of alcohol use. Therefore, if an individual’s momentary impulsivity score was higher than their average score across the data collection period, it was associated with subsequent report of alcohol use. Notably, we also found that individuals with high momentary impulsivity were almost three times more likely to report alcohol use than those with low momentary impulsivity.

Our results align with previous research, providing compelling evidence that impulsivity varies within individuals in response to specific situations [4,42], and at the WS level, variation in impulsivity has been linked to increased alcohol use [4,43]. For example, Griffin (2018) examined momentary aspects of impulsivity (assessed via a four-item scale comprising components of impulsivity in the UPPS) and the relationship with alcohol use in the daily life of adults aged 18 to 45. The findings demonstrated that momentary reported lack of premeditation was a significant predictor of momentary drinking [44]. Similarly, Stamates et al. (2019) found that within-subject variability in impulsivity measured through the momentary impulsivity scale (MIS) was positively associated with increased odds of heavy drinking in a sample of 24 young adults [24]. Although each of these studies employed a different measure of impulsivity, collectively, these findings point to the importance of intra-individual variations in impulsivity within the context of day-to-day experiences. While retrospective self-report questionnaires, such as the DERS, ask participants to report their typical behavior, the mEMA allows for an assessment of behavior over time in daily life [27].

At the BS level, we found a positive association between individual momentary impulsivity scores and subsequent momentary reports of marijuana use. A direct comparison of these findings with other empirical studies is challenging, given that the bulk of other studies examining impulsivity and substance use focus largely on alcohol use, and the evidence surrounding other substances is less abundant [45]. Additionally, there is a dearth of studies examining impulsivity and marijuana use in pregnant and postpartum individuals. However, a recent meta-analysis of 38 studies on impulsivity and marijuana use in adolescents concluded that impulsivity-related traits were associated with marijuana use behaviors [46]. Similarly, in a sample of 109 individuals aged 15 to 26, impulsive traits were associated with problematic marijuana use [47]. A recent study of military veterans also found that certain facets of impulsivity were related to cannabis use problems [48]. While our findings align with these studies, it is difficult to draw direct comparisons due to differences in study populations. The lack of studies in pregnant and postpartum individuals is concerning, given the legalization of marijuana use across the United States and the possible adverse outcomes for infants prenatally exposed to marijuana [49,50].

No associations between momentary impulsivity and cigarette smoking at either the BS or WS levels were observed in our sample. However, a recent systematic meta-review of impulsivity in addictive behaviors concluded that tobacco use appeared to be linked to mild impulsivity [45]. Our study only examined any use of nicotine and did not examine the intensity of use, but a meta-analysis of 97 studies of adults in the general population did find that impulsive traits were positively associated with smoking status [51]. Our findings may be disparate from other published findings, given our measure of impulsivity and the focus on pregnant and postpartum women.

Taken together, these findings provide further evidence of the association between impulsivity and alcohol or marijuana consumption in pregnant and postpartum women. Approximately 45% of U.S. pregnancies are unplanned [52], and impulsive tendencies may play a role [53]. Godiwala and colleagues (2015) reported that 51% of women who scored high in non-planning impulsivity (hasty decision making when planning for the future) reported an unplanned pregnancy compared to 32% of women with a low score in non-planning impulsivity. Unplanned pregnancy has also been associated with unhealthy behaviors, such as alcohol consumption before and during pregnancy. A recent systematic review and meta-analysis of the literature on pregnancy intention and smoking or alcohol consumption found that women with unplanned pregnancies are more likely to report cigarette smoking and alcohol use before and during pregnancy compared to women with planned pregnancy [54]. Therefore, impulsivity may represent a common underlying factor in these at-risk pregnancies, and screening for emotion regulation capabilities, and impulsivity in particular, may represent an important touchpoint in preconceptual and prenatal care. Equally importantly, impulsivity may be a modifiable target for future prevention and treatment interventions to improve outcomes in mothers and infants exposed to substances prenatally.

Ultimately, COVID-19 has served to further exacerbate many adverse health outcomes, particularly the mental health of pregnant and postpartum individuals. Therefore, there is an urgent need to synthesize the growing body of evidence to increase awareness of the continued consequences of the pandemic, to inform scalable strategies to mitigate long-term consequences of the pandemic and to plan for possible future scenarios. For example, there is a need to address factors such as access to care, including development of community-based organizations, which involve individuals who represent the voice of the community and ensure diversity in order to achieve sustainable results. Furthermore, the pandemic has shown that there is an opportunity to leverage novel technologies, such as EMA applications and digital sensors, to increase individual self-awareness of health and wellness through monitoring of behavioral and biological parameters. For example, using EMA applications and digital sensors to promote understanding of personal responses to stress is important to developing resilience to future stress-evoking circumstances [8]. Finally, observational studies of pregnant and postpartum women during the COVID-19 pandemic suggest that social support attenuated the effect of pandemic-related stress on outcomes. For example, in a sample of 77 pregnant participants, perceived social support moderated the association between perceived stress and stress related to childcare during the pandemic [55]. Similarly, in a sample of 593 postpartum participants, Howard and colleagues (2022) found that social support offered protection against the development of depression as well as risky behaviors, such as alcohol and other substance use, during stressful times. Thus, increasing the social support available to pregnant and postpartum women represents an important future focus for healthcare providers.

Several study limitations should be noted. First, a large portion (53.6%) of study participants identified as Hispanic/Latina; therefore, generalizability to other racial/ethnic groups may be limited. Second, while the DERS questionnaire is widely used and validated, certain impulsive traits or behaviors may not be captured by the DERS impulse sub-scale. For example, the DERS impulse sub-scale assesses an individual’s difficulties remaining in control of their behavior when experiencing negative emotions. By contrast, the UPPS-P impulse behavior scale has five sub-scales, which correspond to personality characteristics associated with impulsivity, including positive urgency (tendency to act impulsively in response to positive affect), negative urgency (tendency to act impulsively when experiencing negative affect), lack of premeditation, lack of perseverance and sensation seeking [56]. Because impulsivity is multi-dimensional, future research should extend this research to include other possible dimensions [27]. Another consideration is that impulsivity and substance use were assessed since the last mEMA session. Given that bidirectional relationships between impulsivity and substance use have been proposed [4], we cannot make firm conclusions regarding direct relationships between impulsivity and substance use. Future research would benefit from inclusion of assessments of impulsivity during specific self-reported instances of substance use. Furthermore, this study examined the relationship between reports of impulsivity and substance use while adjusting for pregnancy status and COVID-19-related stress. However, the mediating factors not considered in this study may have influenced this relationship [57]. Yet, mEMA is unique In facilitating the assessment of repeated measures within the context of a pregnant/postpartum individual’s day-to-day living, thus furthering our understanding of the complex challenges that pregnant and postpartum women face.

## 5. Conclusions

Our findings continue to shine a light on the detrimental effect of the COVID-19 pandemic on stress levels and the mental health of pregnant and postpartum women. Learnings from the pandemic—a time of extreme stress—can be used to plan for similar types of events. Additionally, our findings extend the literature regarding emotion regulation, impulsivity and substance use in pregnant and postpartum women. Evidence suggests that there are adverse consequences of impaired emotion regulation capabilities for pregnant individuals and their infants, including increased risk of maternal substance use [58]. Consistent with this literature, we showed that intra-individual variation in momentary impulsivity was linked to increased substance use in pregnant and postpartum women. The present findings highlight the potential implications of emotion regulation difficulties, and impulsivity in particular, for pregnant and postpartum women. Given the evidence that impulsivity is dynamic, varying in given situations, and is likely modifiable during pregnancy [59], there is an urgent need for longitudinal research using novel ecological assessment techniques, which will inform the screening and prevention strategies to mitigate the risk of prenatal substance-exposed pregnancies.

## Figures and Tables

**Table 1:** Sociodemographic characteristics of the study population (*N* = 69).

Characteristics	N (%)
Maternal age (years), Mean + SD	30.0 ± 5.2
Maternal ethnicity:	
Hispanic/Latina	37 (53.6%)
Non-Hispanic/Latina	32 (46.4%)
Maternal race:	
White	48 (69.6%)
African American	2 (2.9%)
American Indian	4 (5.8%)
Asian American	3 (4.3%)
More than one race/Other race	10 (14.5%)
Race not reported	2 (2.9%)
Marital status:	
Single/Separated/Divorced	13 (18.8%)
Married/Cohabitating	56 (81.2%)
Maternal education:	
Less than high school	15 (21.7%)
High school/Some college	17 (24.6%)
College/Professional degree	37 (53.6%)
Currently employed	46 (66.7%)
Pregnancy status:	
Pregnant	49 (71.0%)
Postpartum	20 (29.0%)
COVID-19 and stress levels	
Worsened them significantly	8 (11.6%)
Worsened them moderately	43 (62.3%)
No change	16 (23.2%)
Improved them moderately	2 (2.9%)
Overall level of stress related to COVID-19	
1 (Nothing)	5 (7.2%)
2	7 (10.1%)
3	13 (18.8%)
4	15 (21.7%)
5	19 (27.5%)
6	6 (8.7%)
7 (Extreme)	4 (5.8%)

**Table 2:** Logistic regressions—Baseline impulsivity and any substance use in the past 30 days *(N* = 69).

	Beta Coefficient Estimate	Standard Error	*p*-Value	Odds Ratio	95% CI
**Any Alcohol Use**					
Baseline Impulsivity	0.24	0.14	0.08	1.27	0.97–1.65
Pregnant vs. Not	−2.08	0.60	<0.001	0.02	0.001–0.16
Overall COVID-19 stress	0.30	0.29	0.30	1.35	0.77–2.36
**Any Marijuana Use**					
Baseline Impulsivity	0.14	0.10	0.16	1.15	0.94–1.41
Pregnant vs. Not	−0.26	0.41	0.52	0.60	0.12–2.92
Overall COVID-19 stress	0.04	0.25	0.86	1.05	0.65–1.69
**Any Nicotine Use**					
Baseline Impulsivity	−1.33	0.94	0.16	0.27	0.04–1.67
Pregnant vs. Not	−0.28	0.55	0.61	0.57	0.07–4.97
Overall COVID-19 stress	0.06	0.32	0.86	1.06	0.56–2.00

Note. The baseline DERS impulse scale score is standardized, so that a beta coefficient estimate relates to one-unit increase above the mean score for 69 individuals.

**Table 3:** Association between mEMA impulsivity ^1^ and subsequent mEMA reported substance use.

*Effect*	*Estimate*	*Standard Error*	*p-Value*
**Alcohol Use (89 instances of use; 2110 without use)**			
**Impulsivity**			
Between	0.44	0.31	0.15
Within	0.08	0.04	0.04
Pregnant vs. Not	−7.20	1.34	<0.001
Overall COVID-19 stress	0.20	0.42	0.63
**Marijuana Use (46 instances of use; 2152 without use)**			
**Impulsivity**			
Between	1.25	0.61	0.04
Within	0.02	0.04	0.70
Pregnant vs. Not	−5.68	3.83	0.14
Overall COVID-19 stress	−0.93	1.11	0.40
**Nicotine Use (58 instances of use; 2141 without use)**			
**Impulsivity**			
Between	0.18	0.17	0.28
Within	−0.01	0.19	0.95
Pregnant vs. Not	−1.08	1.07	0.31
Overall COVID-19 stress	0.11	0.35	0.74

Note: There were a total of 2199 observations; ^1^ DERS impulse scale was used to assess impulsivity.

**Table 4:** Association between mEMA impulsivity modeled as binary cut-off ^1^ and subsequent mEMA reported substance use.

*Effect*	*Estimate*	*Standard Error*	*p-Value*	*OR*	*OR 95% CI*
**Alcohol Use (89 instances of use; 2110 without use)**					
Impulsivity ≥ 10	1.02	0.39	0.01	2.77	1.28–6.00
Pregnant vs. Not	−6.71	1.21	<0.001	0.00	0.00–0.01
Overall COVID-19 stress	0.39	0.34	0.26	1.47	0.75–2.89
**Marijuana Use (46 instances of use; 2152 without use)**					
Impulsivity ≥ 10	0.36	0.92	0.69	1.44	0.24–8.73
Pregnant vs. Not	−5.40	2.19	0.01	0.01	0.00–0.33
Overall COVID-19 stress	0.09	0.60	0.89	1.09	0.34–3.52
**Nicotine Use (58 instances of use; 2141 without use)**					
Impulsivity ≥ 10	0.48	0.63	0.44	1.62	0.47–5.53
Pregnant vs. Not	−0.98	0.99	0.32	0.38	0.06–2.61
Overall COVID-19 stress	0.18	0.37	0.61	1.20	0.59–2.47

^1^ DERS impulse sub-scale ≥ 10 vs. <10.

## Data Availability

Data are available at the National Institute of Mental Health Data Archive (NDA). https://nda.nih.gov/.
